# An Algorithm for Task Allocation and Planning for a Heterogeneous Multi-Robot System to Minimize the Last Task Completion Time

**DOI:** 10.3390/s22155637

**Published:** 2022-07-28

**Authors:** Abhishek Patil, Jungyun Bae, Myoungkuk Park

**Affiliations:** 1 Department of Mechanical Engineering-Engineering Mechanics, Michigan Technological University, Houghton, MI 49931, USA; apatil5@mtu.edu (A.P.); mkpark@mtu.edu (M.P.); 2 Department of Applied Computing, Michigan Technological University, Houghton, MI 49931, USA

**Keywords:** multi-robot systems, task allocation, path planning, autonomous navigation

## Abstract

This paper proposes an algorithm that provides operational strategies for multiple heterogeneous mobile robot systems utilized in many real-world applications, such as deliveries, surveillance, search and rescue, monitoring, and transportation. Specifically, the authors focus on developing an algorithm that solves a min–max multiple depot heterogeneous asymmetric traveling salesperson problem (MDHATSP). The algorithm is designed based on a primal–dual technique to operate given multiple heterogeneous robots located at distinctive depots by finding a tour for each robot such that all the given targets are visited by at least one robot while minimizing the last task completion time. Building on existing work, the newly developed algorithm can solve more generalized problems, including asymmetric cost problems with a min–max objective. Though producing optimal solutions requires high computational loads, the authors aim to find reasonable sub-optimal solutions within a short computation time. The algorithm was repeatedly tested in a simulation with varying problem sizes to verify its effectiveness. The computational results show that the algorithm can produce reliable solutions to apply in real-time operations within a reasonable time.

## 1. Introduction

The applications for heterogeneous multi-robot systems (MRS) are increasing thanks to the fast advances in autonomy and artificial intelligence in recent decades [1,2,3,4,5,6]. However, it is still difficult to overcome some of the limitations of current technologies due to the dynamic and unpredictable nature of the world. For the systematic operations of MRS to accomplish complex tasks, four main topics should be resolved: (1) task decomposition, (2) coalition formation, (3) task allocation, and (4) task execution/planning and control [7]. These topics are correlated to each other, which makes the problems even more challenging to solve. The heterogeneity of MRS significantly increases the complexity while causing more layers to the operating system with respect to task decompositions and allocations [8].

Generally, heterogeneity is categorized into either structural or functional heterogeneity. Structural heterogeneity typically includes differences in the robot designs, for instance, differences in motion constraints, running speed, yaw rate, and fuel capacity. On the other hand, functional heterogeneity includes differences in the functions, such as different types of data coming from various sensors, maximum payloads, and the ability to collect samples. Sensor-related issues on each robot are one of the factors that make task allocation and planning more challenging, and there are several active ongoing research inquiries into this [9,10,11,12]. The more heterogeneity factors, the greater the computational load to find an efficient operational strategy for MRS.

This paper focuses on solving a task allocation and planning problem for an MRS with structural heterogeneity. Although it is desirable to consider various heterogeneity factors, we deal with a problem that includes the structural heterogeneity of MRS at this time. Specifically, we assume that the system has heterogeneous mobile robots in a 2D space, such as autonomous surface vessels or ground robots, with different motion constraints and running velocities. We are interested in finding paths for the robots that complete all of the given tasks within the minimum period with a given system. When the robots depart from distinctive locations, and the travel costs do not guarantee symmetricity, we call the problem a min–max multiple depot heterogeneous asymmetric traveling salesperson problem (MDHATSP). This problem is a generalized TSP, which means that it is an NP-hard problem [13]. As preliminary research, two robot problems (2DHTSP, 2DHATSP) were studied in [14], and the problem for multiple structurally heterogeneous robots with symmetric travel costs (MDHTSP) was studied in [15]. At this time, we relax the symmetric travel cost condition by assuming the robots to be Dubin’s vehicles [16] with different minimum turning radii. To focus on the practical aspects of the approach, we put our efforts into lightening the computational loads for large problems while maintaining good solution qualities.

As research on the task allocation of MRS has become more active than in previous years, some publications are dealing with similar problems. However, as is characteristic of MRS operational research, each publication deals with its specific scenario, which makes it difficult to deploy to other scenarios. In a recent publication, Sun and Escamilla proposed an unscented transform-based approach for a task allocation process with uncertainty in situational awareness in [17]. While dealing with functional heterogeneity, they proposed a Hungarian algorithm by focusing on handling uncertainties. Li et al. presented a hybrid large-neighborhood search algorithm that solves a multiple depot autonomous aerial vehicle (AAV) routing problem [18]. The article is focused on addressing an open constraint on return depots without considering heterogeneity. Similarly, Cho et al. presented a sampling-based tour generation algorithm for multiple AAVs by formulating the problem into a generalized MDHATSP [19]. While [19] dealt with the most similar problem to that of this paper, their objective is min-sum, and there is a constraint that the robots must return to one of the terminal nodes. A decentralized auction algorithm for the task allocation of MRS under a limited communication range with a min-sum objective has been proposed in [20]. The task allocation problem of autonomous underwater vehicles’ problems with time and resource constraints and a min–max objective is dealt with in [21]. In [22], an ant colony algorithm for a min–max MDTSP without heterogeneity is proposed, and the results are compared with those of a linear program (LP)-based algorithm. While these approaches deal with the task allocation for MRS, they have distinct objectives and constraints, and none deal with the same problem as that of this paper. This paper aims to fill this gap by targeting the production of reasonable solutions within a short time for a generalized problem.

This paper has several unique contributions as an extension of the preliminary work presented in [15]. The heuristic in [15] is developed for a min–max MDHTSP, which only solves problems with symmetric costs. This paper’s novelty is based on the following contributions: First, we present a new approach in Algorithm 1 for deciding on the dual variables $W_{k}$, which play a role as the weights on travel costs for each robot. Due to generalized travel costs, the algorithm is designed to embrace the asymmetricity of the costs. In addition, new pruning steps for the primal–dual heuristic were developed in Algorithm 2 to enhance the task distribution between the robots. The algorithms are implemented and compared with LP solutions with relaxed integer constraints, the LP rounding method, and our work on a min-sum MDHATSP [23] to verify the effectiveness of the proposed algorithm from the perspective of workload balancing. Lastly, the real-world experimental results are added to verify the feasibility of the algorithm in the field.

The remainder of this paper is structured as follows: In Section 2, we specify the problem and present the formulations. Section 3 presents the primal–dual heuristic approach for a min–max MDHATSP. We present the computational results in Section 4 and, finally, conclude in Section 5.

## 2. Problem Description and Formulation

This section specifies the problem of allocating tasks between robots in a given heterogeneous MRS visiting a given set of targets. In the end, we aim to find a path for each robot that satisfies its motion constraints and completes all of the given tasks by the MRS while minimizing the maximum travel cost among the agents. We assume that the travel costs are asymmetric but still satisfy triangle inequalities. The robots depart from distinct locations and return to their depots once they have completed the assigned tasks. We assume that the robots have different running velocities and minimum turning radii, as briefly introduced earlier. The travel cost is defined as the travel time of the robot and is calculated by $cost_{ij}^{k}=d_{ij}^{k}÷v_{k}$, where $d_{ij}^{k}$ represents the distance of the shortest path from vertex *i* to *j* for robot *k*, and $v_{k}$ represents the average running velocity of robot *k*. We also assume that each robot is labeled as their running velocities decrease and the minimum turning radius increases as their indices increase. Under this assumption, all travel costs will monotonically increase based on their indices, i.e., $cost_{ij}^{1}\leq cost_{ij}^{2}\leq ,\cdots ,\leq cost_{ij}^{m},\;\forall \left\{i,j\right\}\in V_{k},\;k=1,\cdots ,m$.

Given a set of *m* robots and *n* targets, the parameters and decision variables used in the formulation are described as follows:
Parameters:
$D=\{d_{1},\cdots d_{m}\}$A set of depots$T=\{t_{1},\cdots ,t_{n}\}$A set of targets$V_{k}=\left\{\left\{d_{k}\right\}\cup T\right\}$A set of vertices for the *k*th robot$E_{k}=\left\{\left(i,j\right),\;\forall i,j\in V_{k}\right\}$A set of edges that connect all vertices in $V_{k}$$cost_{ij}^{k}$The travel cost of the edge from vertex *i* to vertex *j* for the *k*th robot$\delta_{k}^{+}\left(S\right)$The subset of the edges of $E_{k}$ that enter *S* from $V_{k}\setminus S$


Decision variables:



$x_{ij}^{k}$

the decision variable that represents whether the edge (i,j) is used for the tour of the *k*th robot


$x_{ij}^{k}=\left\{\begin{array}{c} 1\;\mathrm{if}\;\mathrm{the}\;\mathrm{edge}\;\left(i,j\right)\;\mathrm{is}\;\mathrm{traveled}\;\mathrm{by}\;\mathrm{the}\;k\mathrm{th}\;\mathrm{robot}\\ 0\;\mathrm{otherwise} \end{array}\right.$



$z_{U}^{k}$

the decision variable that represents the assignment of targets in *T* for the *k*th robot


$z_{U}^{k}=\left\{\begin{array}{cc} 1 & \mathrm{if}\;U\;\mathrm{contains}\;\mathrm{all}\;\mathrm{vertices}\;\mathrm{not}\;\mathrm{assigned}\;\mathrm{to}\;1\mathrm{st},\;\cdots ,\;k\mathrm{th}\;\mathrm{robot}\\ 0 & \mathrm{otherwise} \end{array}\right.$


*q*
the maximum travel cost among the given *m* robots

Based on the provided parameters and decision variables, the formulation for a linear program (LP) relaxation of the problem is shown below:

(1) $$\begin{array}{cc} C_{LP} & =\min q \end{array}$$

(2) $$\begin{array}{cc} \sum_{\left(i,j\right)\in \delta_{1}^{+}\left(S\right)}x_{ij}^{1} & \geq 1-\sum_{T⊇U⊇S}z_{U}^{1}\;\forall S\subseteq T, \end{array}$$

(3) $$\begin{array}{cc} \sum_{\left(i,j\right)\in \delta_{k}^{+}\left(S\right)}x_{ij}^{k} & \geq \sum_{T⊇U⊇S}\left(z_{U}^{k-1}-z_{U}^{k}\right)\;\forall S\subseteq T,\;k=2,\cdots ,m-1, \end{array}$$

(4) $$\begin{array}{cc} \sum_{\left(i,j\right)\in \delta_{m}^{+}\left(S\right)}x_{ij}^{m} & \geq \sum_{T⊇U⊇S}z_{U}^{m-1}\;\forall S\subseteq T, \end{array}$$

(5) $$\begin{array}{cc} q & \geq \sum_{i,j\in V_{k}}cost_{ij}^{k}\;x_{ij}^{k}\;k=1,\cdots ,m, \end{array}$$

(6) $$\begin{array}{cc} x_{ij}^{k} & \geq 0\;\forall i,j\in V_{k},\;k=1,\cdots ,m, \end{array}$$

(7) $$\begin{array}{cc} \;z_{U}^{k} & \geq 0\;\forall U\subseteq V_{k},\;k=1,\cdots ,m-1, \end{array}$$

(8) $$\begin{array}{cc} q & \geq 0. \end{array}$$

To have the formulation in an LP form, we made the objective to be minimizing *q* as presented in (1), and *q* represents the maximum travel cost, as shown in (5). As the travel costs can be asymmetric, we focused on finding a heterogeneous directed spanning tree by relaxing the outgoing degree constraints while keeping the entering degree constraints in (2)–(4). The non-negativity constraints for the decision variables are (6)–(8). The dual problem of the LP relaxation is derived as follows to develop a primal–dual heuristic:

(9) $$\begin{array}{cc} C_{dual} & =\max\sum_{S\subseteq T}Y_{1}^{+}\left(S\right) \end{array}$$

(10) $$\begin{array}{cc} \sum_{S:e\in \delta_{k}^{+}\left(S\right)}Y_{k}^{+}\left(S\right) & \leq W_{k}cost_{ij}^{k}\;\forall i,j\in V_{k},\;k=1,\cdots ,m, \end{array}$$

(11) $$\begin{array}{cc} \sum_{S\subseteq U}Y_{k}^{+}\left(S\right) & \leq \sum_{S\subseteq U}Y_{k+1}^{+}\left(S\right)\;\forall U\subseteq T,\;k=1,\cdots ,m-1, \end{array}$$

(12) $$\begin{array}{cc} \sum_{k=1,\cdots ,m}W_{k} & \leq 1 \end{array}$$

(13) $$\begin{array}{cc} Y_{k}^{+}\left(S\right) & \geq 0\;\forall S\subseteq T,\;k=1,\cdots ,m, \end{array}$$

(14) $$\begin{array}{cc} W_{k} & \geq 0\;k=1,\cdots ,m. \end{array}$$

In this formulation, $W_{k}$ can be interpreted as the weights that prioritize the robots based on their capabilities, and $Y_{k}^{+}\left(S\right)$ can be considered the price that the vertices in set *S* are willing to pay to be connected to the depot $d_{k}$.

## 3. A Heuristic for Min–Max MDHATSP

The heuristic for a min–max MDHATSP consists of two main procedures, which are presented in Algorithms 1 and 2. While Algorithm 1 focuses on determining the $W_{k}$ values for each robot to improve the workload distribution, Algorithm 2 produces a feasible task allocation and path planning solution for the fixed $W_{k}$ values. In Algorithm 1, we try to transfer the workloads from the robot with the maximum travel cost to other robots to reduce the maximum travel cost in each iteration. In the heuristic presented in Algorithm 2, we used the dual problem (9)–(14) to find a heterogeneous directed spanning forest (HDSF). The resulting forest will become the allocation of the tasks. As we have mentioned, the algorithms treat $W_{k}$ similarly to the weights on robots to prioritize based on their capabilities. $Y_{k}^{+}\left(S\right)$ is treated as the prices that all targets in *S* are willing to pay to become reachable from $d_{k}$. The weighted costs should satisfy the monotonic increase inequalities, $W_{1}cost_{ij}^{1}\leq W_{2}cost_{ij}^{2}\leq \cdots \leq W_{m}cost_{ij}^{m}\;\forall i,j\in T$, all of the time in order to guarantee the feasibility of the algorithm. The notations below are utilized to present the algorithm’s details.
Algorithm Notations:
$F_{k}$A set of edges added to the graph for the *k*th robot$C_{k}$A collection of vertex sets in the graph for the *k*th robot$Y_{k}\left(S\right)$The dual variable of set *S* for the *k*th robot$active_{k}\left(S\right)$The variable that represents the status of $Y_{k}\left(S\right)$
$active_{k}\left(S\right)=\left\{\begin{array}{c} 1\;\mathrm{if}\;\mathrm{set}\;S\;\mathrm{can}\;\mathrm{increase}\;\mathrm{its}\;\mathrm{dual}\;\mathrm{variable}\\ 0\;\mathrm{otherwise} \end{array}\right.$$Cost=\{Cost_{1},\cdots ,Cost_{m}\}$A set of costs for all robots$W=\{W_{1},\cdots ,W_{m}\}$A set of all $W_{k}$$Tour=\{Tour_{1},\cdots ,Tour_{m}\}$A set of assigned paths$TourCost=\{TourCost_{1},\cdots ,TourCost_{m}\}$A set of travel costs of $Tour_{k}$

The algorithm starts with setting the $W_{k}$ values to an equally distribution. This step ensures that at least one feasible solution is produced for the problem as it should satisfy the monotonically increasing inequalities in the default. Once the algorithm finds a feasible solution, it iteratively runs a primal–dual heuristic (Algorithm 2) while changing the $W_{k}$ values to reduce the maximum travel cost. To satisfy the monotonically increasing inequalities of the weighted costs, we designed the algorithm such that the $W_{k}$ also satisfies the monotonically increasing inequalities, i.e., $W_{1}\leq W_{2}\leq \cdots \leq W_{m}$. $W_{k}$ is adjusted with a small amount ϵ (heuristically determined by the user) to share the overloaded work with other robots while remaining tight (12).
**Algorithm 1** A Heuristic for a Min–Max MDHATSP1:$W_{k}=1/m$ for k=1,⋯,m;2:[TourCost,Tour]=GetPartition(Cost,W)3:G←max(TourCost)4:**while** there is no improvement in *G* **do**5:   $\left[TourCost_{k},k\right]=max\left(TourCost\right)$6:   **if** k>1 **then**7:     **for** j = 1:k−1 **do**8:        $W_{j}=W_{j}-\epsilon$9:     **end for**10:     **for** j= k:*v* **do**11:        $W_{j}=W_{j}+\epsilon$12:     **end for**13:   **else**14:     break,15:   **end if**16:   $W_{k}=\frac{W_{k}}{\sum_{k=1}^{m}W_{k}}$ for k=1,⋯,m17:   [TourCost,Tour]=GetPartition(Cost,W)18:   **if** G<max(TourCost) **then**19:     G←max(TourCost)20:   **end if**21:**end while**22:**return** TourCost,Tour

With every fixed $W_{k}$ value, the task assignment is determined by Algorithm 2. Each robot has its own graph, with all targets and a depot as the vertices. Initially, each vertex is in its own set, all dual variables are zero, and the edge set $F_{k}$ is empty. For every iteration, the algorithm search for the dual variable that can make one of the constraints (10) tight with the smallest increment. Add the corresponding edge $e_{k}$ to $F_{k}$. Then, we look into the graph for this robot and check if any valuable changes have been made. First, if a new strongly connected component is formed, but the new component is not reachable from $d_{k}$, then let the new component be an active set. Second, if any set became newly connected to $d_{k}$, then let $d_{k}$ and all reachable sets from $d_{k}$ be a new inactive set. This new component’s subsets should also all be deactivated, while supersets should all be marked. Lastly, if neither the first nor the second happened, deactivate the component. When the algorithm proceeds further, there may be a phase in which there is no active set without entering edges, but some sets are still not connected to the depot. Then, the algorithm generates a new active component for each graph by combining some connected sets with at least one that is marked. The iteration will stop when all sets in the graphs are inactive.
**Algorithm 2** [TourCost, Tour] = GetPartition(Cost,W)1:Initialization 2:$F_{k}\leftarrow \emptyset$, $C_{k}\leftarrow \left\{\left\{v\right\}:v\in V_{k}\right\}$, for k=1,⋯,m3:All vertices are unmarked.4:All dual variables are set to zero.5:$active_{k}\left(\left\{v\right\}\right)\leftarrow 1$, $\forall v\in V_{k}$, for k=1,⋯,m6:$active_{k}\left(\left\{d_{k}\right\}\right)\leftarrow 0$, for k=1,⋯,m  7:Main loop  8:**while** there exists any active component in $C_{1},\cdots ,C_{m}$ **do**9:   **for** k=1,⋯,m **do**10:     Find an edge $e_{k}=\left(i,j\right)\in E_{k}$ with $i\in C_{i},j\in C_{j}$ where $C_{i},C_{j}\in C_{k},C_{i}\neq C_{j}$ that minimizes $\varepsilon_{k}=\frac{W_{k}cost_{ij}^{k}-dual_{k}\left(j\right)}{active_{k}\left(C_{j}\right)}$.11:   **end for**12:   Let the corresponding $C_{j}\in C_{k}$ be $S_{k}$ while $S=\{S_{1},\cdots ,S_{m}\}$ satisfies $S_{1}⊇S_{2}⊇\cdots ⊇S_{m}$ and all are active.13:   $F_{k}\leftarrow \left\{e_{k}\right\}\cup F_{k}$14:   Increase the dual variables of $S_{k}$ with the amount of $\varepsilon_{k}$15:   **if** $e_{k}$ forms a new strongly connected component, and the component is not reachable from $d_{k}$, **then**16:     Let the new strongly connected component be a new *active* component.17:   **else if** $e_{k}$ makes any vertex v∈S reachable from $d_{k}$,18:     Let $d_{k}$ and the all the reachable vertices from $d_{k}$ be a new *inactive* component.19:     **if** k<m **then**20:        Deactivate all subsets of this component in $C_{k+1},\cdots ,C_{m}$.21:     **end if**22:     **if** k>1 **then**23:        Mark all the vertices in the supersets of this component in $C_{1},\cdots ,C_{k-1}$. Deactivate them if the corresponding components consist of all marked vertices.24:     **end if**25:   **else**26:     Deactivate $S_{k}$.27:   **end if**28:   **if** there exists any inactive set without an entering edge that is not connected to the depot, and there exists no $S=\{S_{1},\cdots ,S_{m}\}$ that can be chosen to satisfy the given conditions for any k∈{1,⋯,m}, **then**29:     Pick an inactive component for each *k* consisting of marked vertices with entering or outgoing edges. Combine the connected components until the new component does not have any entering edges. Let the new component be active.30:   **end if**31:**end while** 32:Pruning  33:Let $F_{k}^{\prime }$ be the resulting forest after performing reverse-deleting steps to remove all unnecessary edges.34:Let $P_{k}^{\prime }$ be the vertices in $F_{k}^{\prime }$ for k=1,⋯,m.35:Let $P_{k}$ be the vertices that are only connected to $d_{k}$ for k=1,⋯,m.36:**if** there exist any v∈T that do not belong to any $P_{k}$ for k=1,⋯,m **then**37:   Let $P_{c}$ be a set of such vertices.38:   **while** $P_{c}\neq \emptyset$ **do**39:     Find the closest distances to the depots for all vertices in $P_{c}$.40:     Find the shortest distance. Let $v_{c}$ be the corresponding vertex, and $d_{k}$ be the closest depot.41:     $P_{c}\leftarrow P_{c}\setminus v_{c}$;   $v_{c}\to P_{k}$42:   **end while**43:**else**44:   $P_{k}$ = $P_{k}^{\prime }$45:**end if**46:Find the shortest tour for $P_{k}$ for k=1,⋯,m. 47:**return** TourCost,Tour =0

**Lemma** **1.** *The proposed heuristic produces a feasible plan for the given set of robots such that every given target is visited only once by one of the robots.*

**Proof.**  In Algorithm 2, the main loop terminates when all of the components are inactive. There are only three cases in which the components can be deactivated. First, the component is not any part of the strongly connected components that do not have entering edges, and none of the components’ vertices are reachable from the depots. Second, the component becomes reachable from its depot. Third, one of its supersets/subsets becomes reachable from its depots. As the first condition can deactivate only one component within *S*, the termination condition cannot be met by only the first condition. That means that the second or third condition should meet at least one in order to terminate the main loop, thereby implying that all components should be connected to at least one depot. The pruning steps ensure that each target is connected to only one depot if there exists any target that is connected to multiple depots. Thus, Algorithm 2 produces a feasible solution for the given set of robots such that every given target is visited only once by one of the robots. As Algorithm 1 updates the $W_{k}$ values while maintaining the monotonically increasing inequalities, the proposed heuristic produces a feasible solution to the problem. □

## 4. Implementation Results

### 4.1. Simulation Results

We implemented the heuristic and performed the simulation repeatedly with varying problem sizes to validate the proposed heuristic. All simulations were performed in a PC equipped with an Intel®Core™ i7-7800X CPU running at 3.5 GHz with 64 GB RAM. The numbers of robots and targets varied from 3 to 6 and 20 to 50, respectively. To have a standard for the produced solution qualities, we used the optimal costs for the LP relaxation problem calculated by the commercial software CPLEX [24] as lower bounds. Though we repeated the tests 50 times for each size, we only tested the heuristic for 100 targets to estimate the computational time due to the extensive computation time of LP for large-sized problems. Using the LP solution, We also applied the LP rounding method, which assigns the target to the one with the largest partitioning variable value in order to compare the results based on the calculated LP relaxation costs. In addition, we applied our previous algorithm that solves a min-sum MDHATSP [23] to verify the effectiveness of the algorithm and, specifically, to reduce the last task completion time. The coordinates of depots and targets are randomly generated within a space of 3 m × 3 m with a uniform distribution. As previously mentioned, the robots are labeled as their running velocities decrease while the minimum turning radius increases in order with the index. $cost_{ij}^{k}$ was set to the minimum travel time by calculating the Dubin’s path [16] from *i* to *j* divided by the average running velocity of the *k*th robot. The path within each assignment was generated using LKH [25] for both LP rounding and the proposed heuristic.

**Figure 1:**
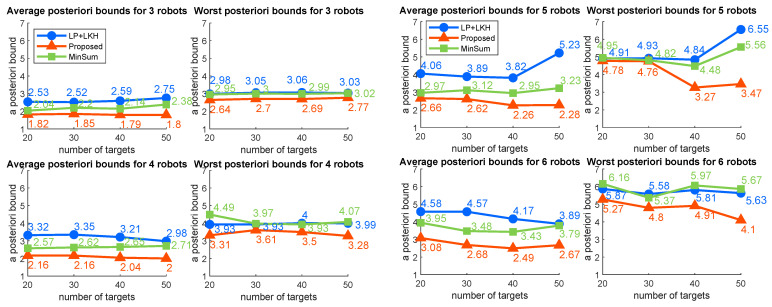
Average (**left**) and worst (**right**) posterior bounds.

The average and maximum posteriori bounds are shown in Figure 1. The posteriori bound has been calculated by $Cost_{algo}÷Cost_{LP}$, where $Cost_{algo}$ represents the cost generated by an algorithm, and $Cost_{LP}$ represents the optimal cost of the LP relaxation problem. As the objective of the problem is min–max, which is nonlinear, the gap between the costs of the original mixed-integer problem and the LP relaxation problem is a bit large, which means that the actual solution qualities are more reasonable than the presented numbers. As we can see from the results, the average posteriori bounds of the proposed heuristic stayed the lowest, while the min-sum heuristic remained in the middle, and the LP rounding method was the highest. The worst posteriori bounds for the proposed algorithm also remained the lowest regardless of the problem sizes.

**Figure 2:**
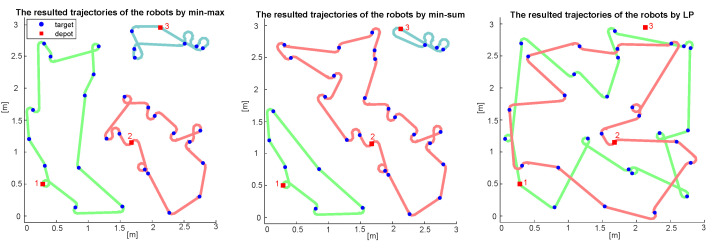
Three solutions were derived from different approaches for 3 robots with 30 tasks. The numbers next to the depots represent the index of the robots. The green, red, and blue paths represent the trajectories for robots 1, 2, and 3, respectively. The last task completion times for the proposed heuristic, min-sum heuristic, and LP rounding method are 8360, 11,445, and 12,309 s, respectively. The computation times for the proposed heuristic, min-sum heuristic, and LP rounding method are 2.43, 0.78, and 6495.11 s, respectively.

**Figure 3:**
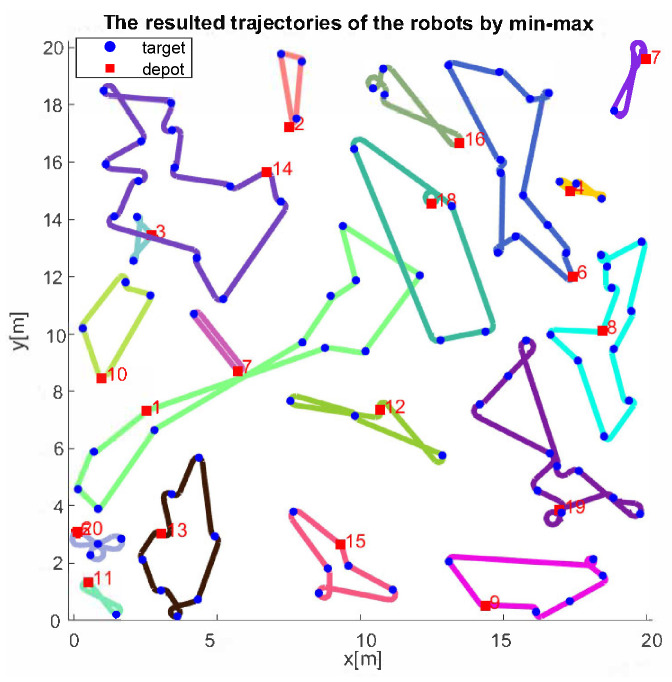
A solution derived by the proposed heuristic for 20 robots with 100 tasks. The numbers next to the depots represent the index of the robots, and the colored path connected to each depot is the trajectory of the corresponding robots. The computation time is 1567 s, which is around 26 min.

The average and maximum computation times are shown in Table 1. Compared to the results of the min-sum heuristic, which was an average of 9 s for six robots and 50 targets, the computation time is longer for min–max cases, with an average of 35 s. For 10 instances of 20 robots and 100 targets, the algorithm produced a solution within an average of 35 min. Considering the fact that the algorithm can handle more generalized problems, the computation time is still within an acceptable range for real-world operations, especially for large-sized problems. Figure 2 shows the results from three different algorithms for an instance of three robots and 30 targets within a 3 m × 3 m space, and Figure 3 shows the results from the proposed heuristic for an instance of 20 robots and 100 targets within a 20 m × 20 m space.

### 4.2. Field Experiments

**Figure 4:**
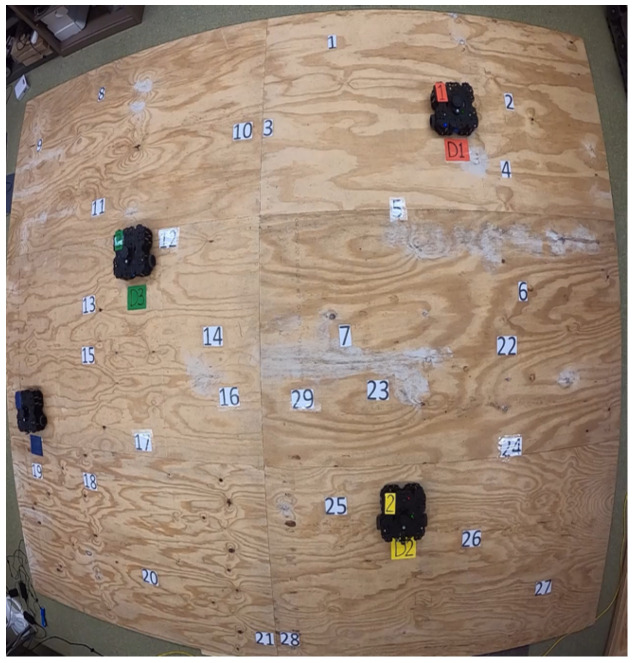
An experimental scene for one of the field experiments. The colored locations represent the depots, and others represent the target locations.

In addition to the simulation, we performed field experiments to verify the effectiveness of the MRS in real-time applications with a small-sized problem. The MRS consists of four ground mobile robots, Turtlebot3 Waffle Pi [26], and each robot has a different limited running velocity. The experiment site is a size of 16 ft × 12 ft and is equipped with an OptiTrack Motion Capture System (with 8 OptiTrack Prime 17W cameras) to transfer the locations of the robots in real-time. The central control system is implemented in ROS [27] to navigate the robots. The experimental setup is shown in Figure 4.

We tested the proposed algorithm for a problem with 29 targets and distinctive depots for the robots. Once the task allocation and the path generation are completed, the robots immediately work on the given tasks by following the provided paths. In our experiments, the linear velocities were set to 0.1, 0.083, 0.071, and 0.063 m/s, respectively, to include the heterogeneity of the system. To verify the effectiveness of the proposed heuristic, we compared the results with our preliminary research, the primal–dual heuristic for min-sum MDHATSP [23].

**Figure 5:**
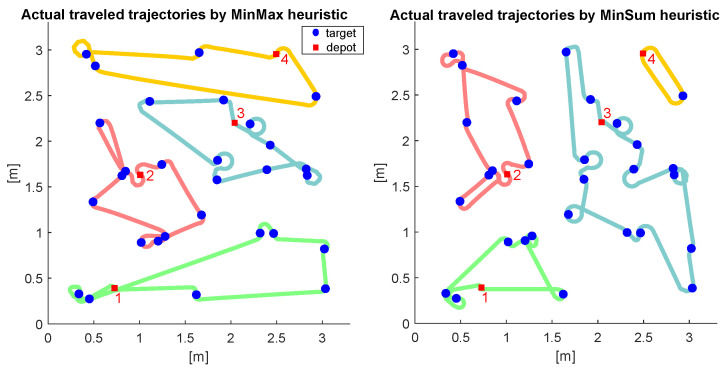
The resultant trajectories of the robots with the proposed algorithm (**left**) and min-sum heuristic (**right**) for an instance with 4 robots and 29 targets. The numbers next to the depots represent the index of the robots. The green, red, blue, and yellow paths represent the trajectories for robots 1, 2, 3, and 4, respectively.

The results from the field experiment results are shown in Figure 5 and Table 2. Figure 5 shows that the robots were able to complete their tasks as provided by the heuristics. As shown in Table 2, though the min-sum heuristic ran in 0.95 s to complete the task allocation and path generation, the workload was overloaded to robot 3, which caused a longer last task completion time. On the other hand, the proposed heuristic ran in 3.23 s, which is a bit longer, but the workload has been well distributed, resulting in a shorter last task completion time. However, the sum of the travel costs was better with [23] than with the proposed algorithm, which makes sense as it aimed to minimize the total travel costs to reduce the complexity of the problem. The results show that the newly proposed approach is practical for the real-time operations of actual applications as the new heuristic deals with a more generalized problem with a better workload distribution within a reasonable computation time.

## 5. Conclusions

This paper proposes a heuristic that efficiently allocates the given tasks among the heterogeneous mobile robots while minimizing the last task completion time. The min–max MDHATSP is a fundamental problem that may arise in many multi-robot systems’ applications. The problem’s formulation, used to design a primal-dual heuristic, is presented, and the details of the algorithm are discussed. Finally, as validation steps, the implementation results are presented to show the effectiveness and potential of the algorithm for use in actual applications. Our future work aims to extend this research to more generalized problems by considering more constraints from the heterogeneity in MRS, such as capacity restrictions or fuel constraints.

## Figures and Tables

**Table 1:** Computation time in seconds.

Tasks	LP Rounding	Min-Sum	Proposed	LP Rounding	Min-Sum	Proposed
	Average with 3 robots			Worst with 3 robots		
20	4.36	0.23	0.68	6.41	0.58	1.95
30	67.39	0.65	1.77	85.97	1.07	3.38
40	619.08	1.46	4.04	888.91	1.83	7.01
50	3614.6	2.83	8.66	4922.4	3.42	12.75
	Average with 4 robots			Worst with 4 robots		
20	5.89	0.38	1.06	8.66	1.47	2.29
30	82.22	1.07	3.30	112.46	1.99	5.93
40	952.54	2.36	7.77	4183.8	3.22	12.92
50	5128.1	4.34	13.66	4434.8	5.11	24.33
	Average with 5 robots			Worst with 5 robots		
20	14.37	0.52	1.60	18.97	1.00	4.16
30	267.74	1.50	4.75	446.77	2.02	9.27
40	2960.1	3.41	11.55	4183.8	3.89	20.96
50	10,920	6.67	23.88	15,875	7.56	39.74
	Average with 6 robots			Worst with 6 robots		
20	14.22	0.69	2.12	17.29	1.53	5.39
30	265.13	2.14	8.00	347.92	2.59	15.97
40	3153.3	4.62	16.79	4434.8	5.52	32.63
50	15,527	8.99	35.05	21,225	10.32	64.30

**Table 2:** Computation and travel time in seconds of the field experiments. The min-max heuristic-based run had the minimum last task completion time, as presented in red, and the min-sum heuristic-based run had the minimum sum of all travel time, as shown in blue.

	Computation	Robot1	Robot2	Robot3	Robot4	Sum of Travel	Last Task Completion
Min–Max heuristic	3.23	83.3	81.5	88.7	**103**	356.5	106.23
Min-Sum heuristic	0.95	56.7	85	**142.7**	24.7	309.1	143.65

## Data Availability

Not applicable.
